# Eye Movement Sequences during Simple versus Complex Information Processing of Scenes in Autism Spectrum Disorder

**DOI:** 10.1155/2011/657383

**Published:** 2011-07-19

**Authors:** Sheena K. Au-Yeung, Valerie Benson, Monica Castelhano, Keith Rayner

**Affiliations:** ^1^School of Psychology Shackleton Building, University of Southampton, Highfield SO17 1BJ, UK; ^2^Department of Psychology, Queen's University, Kingston, ON, Canada K7L 3N6; ^3^Psychology Department, University of California, San Diego, Mandler Hall, La Jolla, CA 92093, USA

## Abstract

Minshew and Goldstein (1998) postulated that autism spectrum disorder (ASD) is a disorder of complex information processing. The current study was designed to investigate this hypothesis. Participants with and without ASD completed two scene perception tasks: a simple “spot the difference” task, where they had to say which one of a pair of pictures had a detail missing, and a complex “which one's weird” task, where they had to decide which one of a pair of pictures looks “weird”. Participants with ASD did not differ from TD participants in their ability to accurately identify the target picture in both tasks. However, analysis of the eye movement sequences showed that participants with ASD viewed scenes differently from normal controls exclusively for the complex task. This difference in eye movement patterns, and the method used to examine different patterns, adds to the knowledge base regarding eye movements and ASD. Our results are in accordance with Minshew and Goldstein's theory that complex, but not simple, information processing is impaired in ASD.

## 1. Introduction

Autism spectrum disorder (ASD) is a neurodevelopmental condition characterised by a triad of impairments in social relationships, communication, and imagination [1]. Along with the diagnostic symptoms in the social domains, individuals with ASD also have an atypical cognitive profile in which they are impaired at complex cognitive tasks [2], with spared or sometimes enhanced lower level perceptual abilities [3]. 

More than a decade ago, Minshew and Goldstein [4] put forward a multiple primary cognitive deficit model which suggests that autism is a disorder of complex information processing across cognitive domains in which the visuospatial system is intact. 

Minshew et al. [5] explained that complex information processing tasks are those which require integration of multiple features rather than reliance on one or two individual features, speed of processing, processing of large amounts of information, or processing of novel material. All of these task demands tax information processing or integration capacity and reveal the limitation in processing in autism (p. 383). 

This is based on findings from an early study in which Minshew et al. [6] administered a neuropsychological battery of tests that examined attention, motor, memory, language, and reasoning to 33 high functioning autistic individuals and 33 individually matched typically developed (TD) controls. It was found that the ASD individuals were intact or superior in tasks involving basic abilities including attention, simple memory, simple language, and the rule learning aspects of abstract reasoning. However, impairments were found selectively in each cognitive domain with the highest information processing demands, including complex motor, complex memory, complex interpretative aspects of language, and reasoning domains. These results were subsequently replicated in another study [7] in 56 high-functioning children with autism using cognitive and neuropsychological assessment measures adapted for children, with the exception of more pronounced impairments in sensory perceptual domains and less reasoning deficits which could be explained by brain maturation mechanisms. 

 The consequences of complex information processing deficits in ASD are that in real-world situations, this population will experience difficulty in fast dynamic social interactions because of their inability to quickly process relevant information. Some of the clinical implications are that information given to individuals with ASD needs to be presented in small chunks and at a slower pace.

Recently, experimental findings from eye movement research have begun to support the complex information processing deficits in ASD. Tracking of eye movements provides a noninvasive measurement of moment-by-moment online cognitive processing involved in different tasks [8, 9]. Therefore, analyses of eye movements in ASD should give an account of what is capturing, maintaining, and driving attention for these individuals in scene inspection. If individuals with ASD are indeed impaired at complex cognitive processing, then their pattern of eye movements should only deviate from that of typically developed individuals for a complex information processing task, but not for simple information processing task, when viewing the same materials.

Benson et al. [2] examined the effect of top-down (social versus material) task instructions during a partial replication of the Yarbus study [10], where participants had to inspect the Repin picture “Unexpected Return”. It was found that TD individuals modulated their eye movements when viewing the scene according to the task instructions they were given prior to inspection. When given a social instruction (Estimate how long the unexpected visitor has been away), they spent more time looking at heads and people in the scene compared to when they were given a material instruction (Estimate the material circumstances of the family). Similarly, when given the material instruction, they spent more time looking at objects in the scene compared to when the social instruction was given. In contrast to TD participants, individuals with ASD did not show such modulation of eye movements, suggesting a deficit in information processing for this task. However, because only “complex” or “top-down” instructions were employed for this study, we do not know whether the ASD group would have modulated their eye movements appropriately for a simple processing instruction. Therefore, in the present study, we systemically manipulated the level of task complexity (simple versus complex) in order to investigate this question.

 In the current paper, we report a follow-up eye movement sequence analysis using a novel technique, on the data from an experiment of which the more standard eye movement measures were already reported previously [11]. In scene perception research, the most commonly used eye movement measures are those which describe where (fixation location) and for how long (fixation duration) a person looks in a scene. These can give an indication of how specific features in a scene capture the attention of a viewer and to what extent the viewer processes these features once they have begun inspection of them. However, to date, few studies have looked into the sequence of eye fixations that individuals produce when examining different parts of a scene. This kind of analysis is important for the reason that it can inform us on how similar two people's scanpaths are over the entire trial period while looking at same scene with the same task at hand. This should potentially give an additional index of sampling similarities or differences that exist for the different type of task instructions used in this experiment for both TD and ASD individuals.

Recently, the development of analysis tool called ScanMatch by Cristino et al. [12], has made it possible to compare directly the sequence (order, location, and duration) of fixations for different individuals completing the same task. The effectiveness of ScanMatch for eye movement sequence analysis has been tested using several simple tasks [12]. In one of these, a participant was shown some red and green numbers scattered on a screen and instructed to look at the numbers of one colour in ascending or descending order. This resulted in four conditions, which were repeated five times each in random order. Each sequence of eye movements for each condition was compared to every other sequence from the same condition and also to every sequence in all other task conditions. In line with Cristino et al.'s prediction, analysis using ScanMatch revealed that comparisons between sequences from the same conditions produced better match scores than comparisons between sequences from different conditions. What this essentially means is that the ScanMatch method is able to give an accurate measurement of similarity for two sets of eye movements. 

In the current study, we investigated sequences of eye movements in ASD and TD participants for two types of task demand: simple versus complex. Both groups of participants viewed complex pairs of pictures under two inspection instructions. In the “spot the difference” task, participants were presented with two pictures and were asked to decide which picture had a detail missing; this is a simple processing task in that the decision required is concrete, requiring only basic visual pattern matching. In the “Which One's Weird” (WOW) task, participants were asked to decide which one of two pictures looked odd, unusual, or weird. This is a complex information processing task as it requires participants to draw upon their prior knowledge to make a novel subjective value judgment. In neuropsychological terms, this involves the integration of top-down information from the higher frontal regions of the brain with bottom-up visual information from the occipital regions. 

 If complex information processing is impaired in ASD as Minshew and Goldstein [4] claimed, this should be reflected in less similar sequences of eye movements between participant groups but more similar sequences within groups using Cristino et al.'s sequence analysis technique. And, as simple perceptual processing should be intact in participants with ASD, and there are no differences in the type of search strategy used between individuals with or without ASD [3, 13], we should expect to find that eye movement sequences for our simple processing task should be equally similar within and between participant groups.

## 2. Method

### 2.1. Participants

The TD group was comprised of 13 adolescents and adults aged 19 to 48 years recruited through the School of Psychology intranet in the University of Southampton and from the general public. The ASD group was comprised of 14 adolescents and adults aged 18 to 49 years previously diagnosed with Asperger's syndrome disorder (11) and high functioning autism (3), using standardized diagnostic instruments. They were recruited from the Southampton Adult Asperger's Society, the University of Southampton, and the Hampshire Autistic Society and the Autism Diagnostic and Research Centre (ADRC). Participants were group-matched in age, verbal IQ, performance IQ, and full-scale IQ. All participants completed the 50-items Autism-Spectrum Quotient (AQ) questionnaire [14]. Higher AQ scores imply more autism-like traits. Participants in the ASD group scored significantly higher than participants in the TD group, *t*(25) = 6.25, *P* < .001, which confirmed that the ASD group displayed disproportionately more autism-like traits than the TD group. Participants' demographics are summarised in Table 1.

### 2.2. Stimuli

**Figure 1:**
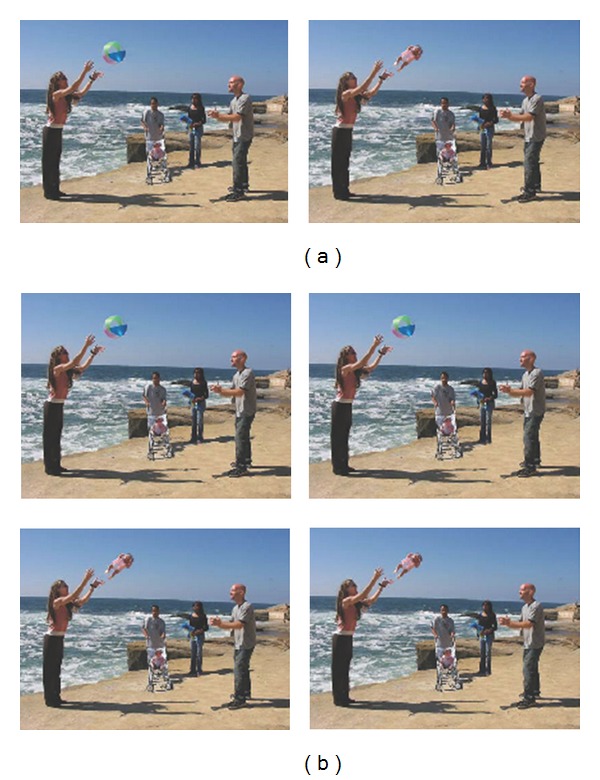
(a) Example stimuli for the WOW task. Picture on the left is the “normal” picture. Picture on the right is the “weird” picture. This picture was digitally manipulated in which the beach ball in midair is replaced by a baby. (b) Example stimuli for the STD task. The stimuli pair could either be two normal pictures (top two) or two “weird” pictures (bottom two). The detail missing pictures are shown on the right of the picture pairs; the shadow of the woman in the background was digitally removed.

For the complex information processing (which one's weird-WOW) task, participants viewed 28 pairs of digitally manipulated complex scenes presented side by side on a display monitor (see Figure 1(a)). For each pair, the pictures were identical apart from a “weird” feature in one of them. Before the start of the experiment, participants were instructed to “press the left button on the button press controller if the picture that looks weird is on the left” and “press the right button if it is on the right”. The left-right location of the weird and normal pictures was counterbalanced.

For the simple information processing (spot the difference-STD) task, participants viewed 28 pairs of digitally manipulated complex scenes. For each pair, the two pictures were identical apart from a detail that is missing in one of them. Before the start of the experiment, participants were instructed to “press the left button on the button press controller if the picture with the detail missing is on the left” and “press the right button if it is on the right”. The left-right location of the detail missing pictures was counterbalanced. For each stimuli set, the pictures pairs could both either be weird or normal, and this was also counterbalanced (see Figure 1(b)). 

The order at which the stimuli set were presented was randomized. The first four pairs of pictures in each task condition were practice trials, resulting in 24 experimental trials for each task condition. Examples of the stimuli can be seen in Figure 1, and the complete set of picture pairs used for both tasks can be obtained from the first author of this paper.

### 2.3. Apparatus

Participants viewed the stimuli binocularly on a SonicView P227f 21 in monitor with a resolution of 1024 by 768 pixels; eye movements were recorded monocularly at 1000 Hz using an Eyelink 1000 eye tracker (SR Research Ltd, Osgoode, Canada). A chin rest and a forehead support were used to maintain participants' head position at a viewing distance of 60 cm from the monitor. Each participant was calibrated using a nine-point matrix prior to testing.

### 2.4. Procedure

Participants read instructions for the two tasks and were shown sample stimuli on paper before each task began and confirmed that they understood the instructions by telling the experimenter verbally what they thought that they had to do for each task, and by giving the appropriate response. For the eye movement recording, participants were seated in a dark room facing the monitor. The monitor and the eye tracker were interfaced with a computer that controlled the experiment. Upon successful calibration, participants completed the four practice trials and 24 experimental trials. Before the start of each trial, 5 black dots at the top, bottom, left, right, and centre appeared on a gray screen, and participants were asked to look at the central fixation dot. This allowed the experimenter to see whether the eye tracker was capturing the location of participant's fixation accurately and, therefore, recalibrate if necessary. Once participants' point of fixation matched the central dot satisfactorily, the experimenter pressed the space bar to initiate the trial. Each trial ended when participants pressed either the left or right button on the button controller to give their response, or after a 20 seconds time limit. At the end of the experiment, participants were debriefed and paid *£*20. 

### 2.5. Eye Movement Sequence Analysis

#### 2.5.1. Creating Sequences

We adopted the use of ScanMatch, an open source toolbox [12] comprised of a series of analysis algorithm that runs on Matlab, to compare the similarity between participants' sequences of eye movements. A sequence is a string of codes representing regions of interest (ROIs) in the order a participant fixated these during one experimental trial for one condition. Each image containing the stimuli sets was divided into 676 (26 × 26) rectangular ROIs. Each ROI was represented by a combination of two letters (ranging from Aa to Zz) and each fixation within the ROI was tagged with its name in the string sequence. Sequences were created using the X and Y pixel coordinates of each fixation to derive the ROI at which the fixations landed. ScanMatch also allowed temporal binning by repeating the name of the ROI in the sequence proportional to the fixation duration. Our strings sequences were divided into 100 ms bins. The resulting sequence, therefore, incorporates spatial location, sequential information, and temporal duration. For example, if a participant looked sequentially to three locations corresponding to ROIs Aa, Ba, and Cc for 100 ms, 200 ms, and 300 ms, respectively, the eye movement sequence will be represented by AaBbBbCcCcCc.

#### 2.5.2. Sequence Matching

 A sequence alignment algorithm in ScanMatch [12] produces a score that quantitatively describes how similar two sequences are. The algorithm finds the best alignment over the whole string of two sequences by maximising its score. The sequences were aligned based on a substitution matrix which provides a score for every alignment based on the spatial relationship between ROIs. The final score for the goodness of match of two sequences is normalised as the algorithm is dependent on substitution matrix and length of the sequences. The best match of two sequences will give a score of 1.

Each participant's eye movement sequences were matched to the sequences of every other participant within and between participant groups. Only sequences for the viewing of the same stimuli set, within the same counterbalancing block of the same condition were matched. Our analysis produced alignment scores for three types of match: within ASD participant group match (ASD versus ASD), between group match (ASD versus TD), and within TD group match (TD versus TD) for both tasks for all 24 stimuli sets. 

## 3. Results

We present a summary of the measures reported in [11] below, before reporting on the sequence analyses findings for the current paper, in order to make interpretation of the sequence analyses results easier to understand.

### 3.1. Accuracy

There were no differences between ASD and TD participants for the simple STD task (ASD 59%, TD 60%) and for the WOW task (ASD and TD 98%).

### 3.2. Eye Movements

There were no differences for any of the eye movement measures between the ASD and TD groups for the simple STD task. However, for the complex WOW task, the ASD group took longer to begin inspecting the target region (ASD *M* = 1389 ms, TD *M* = 1202 ms), made more fixations before they entered the target region (ASD *M* = 4.1, TD *M* = 3.4), and when they got to the target region, they did not immediately pick up that it was the weird region, as indexed by the first fixation duration (ASD weird *M* = 251 ms, normal  *M* = 245 ms; TD weird *M* = 265 ms, normal *M* = 209 ms); see [11] for more details.

### 3.3. Eye Movement Sequence Analysis

We had two within-subject independent variables: task (2 level: WOW, STD) and type of match (3 levels: ASD versus ASD, ASD versus TD, TD versus TD). As our data set is not based on scores for each participant but on alignment scores between participants, it was not possible to compute the standard participant analyses. Therefore, items analyses were conducted in which we computed mean alignment scores for each of the 24 stimuli sets. 

### 3.4. ANOVA 

As the complex information processing deficits theory predicts that cognitive processing deficits should only appear for complex but not simple task, we expected that eye movement sequences would be dissimilar between ASD and TD participants for the WOW (complex) task and similar for the STD (simple) task. For the WOW task, mean alignment scores should therefore be higher when participants eye movements sequences are matched with those within the same group (ASD versus ASD and TD versus TD) than from a different group (ASD versus TD), whereas for the STD task, there should be no differences in mean alignment scores both within and across groups.

**Figure 2:**
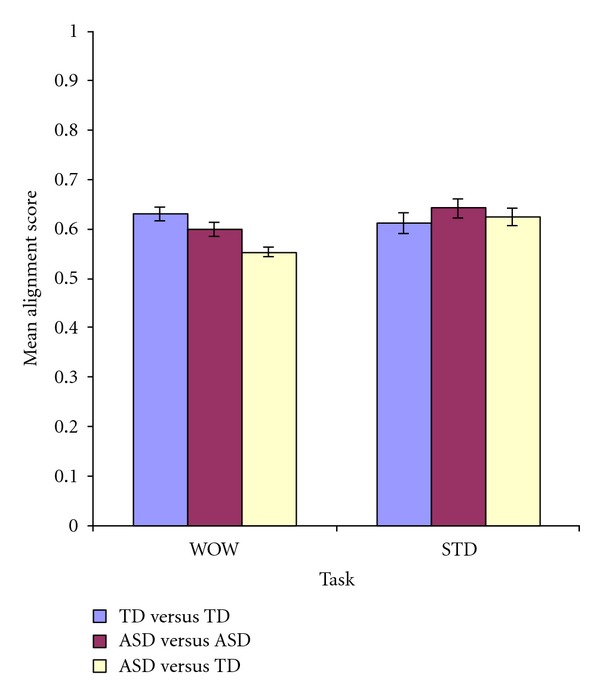
Mean alignment scores (±SE) for the three types of match for each task instruction.

A two-way repeated measure (2 × 3) ANOVA was computed to compare the effect of types of match and task on mean alignment scores. There was a significant main effect of types of match on mean alignment score, *F*(2, 46) = 7.35,  *P* = .002, *np* ^2^ = .242 (ASD versus ASD: *M* = .620, SE = .011; TD versus TD: *M* = .621, SE = .015; ASD versus TD: *M* = .588, SE = .010). The main effect of task on mean alignment score was not significant, *F*(1, 46) = 2.16, *P* = .155, *np* ^2^ = .086. And finally, there was a significant interaction between task and types of match on mean alignment score, *F*(2, 46) = 8.009, *P* = .001, *np* ^2^ = .258 (see Figure 2). What this means is that the significant main effect of types of match on mean alignment scores might be driven by differences within one of the tasks. 

In order to investigate this possibility, six planned pair-wise comparisons were computed with Bonferroni correction (*α* = .05/6 = .008) comparing alignment scores for the three types of match within each task. The critical expected finding is that for the WOW task, mean alignment scores for both ASD versus ASD (*M* = .599, SD = .0766) and TD versus TD (*M* = .630, SD = .0697) were significantly higher than ASD versus TD (*M* = .552, SD = .0555), both *Ps* < .001, demonstrating that eye movement sequences were more different between groups than within groups for the complex information processing task. Although there was a small numerical difference in mean alignment scores between ASD versus ASD and TD versus TD for the WOW task, *P* = .074, this could be accounted for by the slight increase in variability within the ASD group; however, this trend no longer approached significance after Bonferroni correction. Additionally, the three comparisons for the STD task were all nonsignificant (all *Ps* > .1) showing that, as predicted, all participants had similar eye movement sequences for the simple information processing task. Detailed results of all six *t*-tests are shown in Table 2. 

## 4. Discussion

The current study investigated the effect of two different types of task instruction on eye movement sequences for TD and ASD participants when they viewed pairs of complex scenes. If ASD is a disorder of complex information processing where simple or perceptual processing is intact [4], we would predict that individuals with ASD would show processing deficits for the complex but not the simple information processing task. Our results show that the eye movement sequence was similar between the two groups for the simple information processing task but dissimilar for the complex information processing task, suggesting that a processing difference between ASD and TD occurs exclusively for the complex task.

The eye movement sequences findings from the current study are in line with a previous analysis of the eye movement data from the same experiment, where we used more standard eye movement measures [11]. In that analysis, for the simple STD task, we found no between group differences in performance in terms of time taken to respond, time taken to begin fixating in the target region, and number of fixations made before the target region of the missing detail was fixated as well as other more global measures such as total time and mean fixation duration. In contrast for the complex WOW task, ASD participants took longer to make their decision and made more fixations compared to TD participants before they fixated the “weird” target region. Furthermore, a subtle processing difference was observed for the WOW task when we examined the first fixation duration to the target region. Fixation durations give a measure of processing time [8, 9]. Longer first fixation durations show that information is attended to differentially from initial inspection, indicating significance of that information for the task at hand. We found that the TD participants had significantly longer first fixation durations to the target region of the “weird” picture compared to the normal picture and that this difference was absent for participants with ASD. This suggests that the source of difference in the sequence analysis comes from the fact that participants with ASD did not immediately recognize the “weird” feature of the scene when they first fixated it as TD participants did and that more task irrelevant parts of the scene were explored before the correct decision was made. Reduced efficiency of complex information processing in ASD individuals could potentially affect these individuals' ability to react spontaneously during everyday social interaction, accounting for social impairments in ASD. 

Complex information processing deficits in ASD have been linked to disturbance in the neocortical systems [15]. Individuals with ASD were reported to be impaired in eye movement tasks which depend upon higher level voluntary cognitive control of saccades including the oculomotor delayed response task and antisaccade task [16]. Impairments in these tasks are indicative of abnormalities in prefrontal cortex and functional connectivity [17, 18]. Hence, processing deficits in complex tasks in ASD, such as those observed in this study, could result from underdevelopment of higher order cortical regions and connections that subserve these regions of the brain (see Müller [19] for a review). Although it remains to be empirically tested, underdeveloped feedback systems at the level of the cortex may in the future be found to account for increased processing time in ASD for complex information processing tasks.

Some may argue that the large age range of the participants and the relatively small sample size were potential weaknesses of the study. However, we are doubtful that these had any significant effects on the results at all. Firstly, the age range is just as diverse in the TD group as in the ASD group with *t*-tests on the age of the participants revealing no significant differences between the two groups. Furthermore, the two groups were matched in the IQ measures, mismatching only on AQ measures, which is the expected difference between the two groups. Secondly, despite the small sample size, the experimental effects were extremely robust, *P* = .002 for the main effect of types of match on alignment scores, *P* = .001 for the interaction between types of match and task on alignment score, and *P* < .001 for both expected differences in the planned comparisons, showing that eye movement sequences were more different between groups than within groups for the complex task. We are, therefore, confident that increasing the number of participants would simply serve to further support our current results. 

Another limitation of the current study relates to the output of the analysis methodology. The ScanMatch procedure [12] does not tell us where exactly the differences lay between two eye movement sequences, and therefore, in order to say something about specific processing differences, it would be best used in conjunction with more standard eye movement measures. Nevertheless, it has provided us with a useful index of the magnitude of similarity/differences between eye movement sequences for typical individuals and a clinical population for the tasks in this study. 

## 5. Conclusions

The purpose of this study was to carry out a sequence analysis on eye movement data in order to test the complex information processing deficit theory of ASD [4]. This analysis has revealed that individuals with ASD exhibit a different pattern of eye movements compared to TD individuals when viewing pairs of pictures exclusively for a complex task. These results further support impaired complex information processing with intact simple information processing ability in ASD. What remains to be examined are the specific conditions that differentiate between simple and complex information processing in ASD, and eye movement recordings and analyses could be instrumental in establishing those conditions.

## Figures and Tables

**Table 1:** Means, standard deviations, and range of age, verbal IQ, performance IQ, full-scale IQ, and AQ scores for ASD and TD group.

Measures	TD			ASD			*t*
	*M*	SD	Range	*M*	SD	Range	
Age	26.9	7.83	19–48	29.5	10.6	18–49	.734
Verbal IQ	110	14.7	71–125	109	23.0	77–146	.134
Performance IQ	113	13.2	88–131	109	17.7	72–134	.665
Full-scale IQ	113	14.9	76–132	110	21.1	73–139	.403
AQ	15.0	5.63	5–25	32.9	8.81	19–48	6.25*

Note. *significant at *P* < .001.

**Table 2:** Planned comparisons of mean alignment scores between different types of match within each task.

Pair		*t*	*df*	*P*
	WOW task			
ASD versus ASD	ASD versus TD	4.12	23	*
ASD versus TD	TD versus TD	4.92	23	*
TD versus TD	ASD versus ASD	1.87	23	.074

	STD task			
ASD versus ASD	ASD versus TD	1.45	23	.161
ASD versus TD	TD versus TD	.983	23	.336
TD versus TD	ASD versus ASD	1.52	23	.143

Note: *significant at *P* < .001.
